# Editorial: Community series in plant viruses: Molecular plant virus epidemiology and its management, volume III

**DOI:** 10.3389/fmicb.2023.1125486

**Published:** 2023-03-14

**Authors:** Rajarshi Kumar Gaur, Akhtar Ali, Xiaofei Cheng, Bright Ogheneovo Agindotan, Xifeng Wang

**Affiliations:** ^1^Department of Biotechnology, Deen Dayal Upadhyaya Gorakhpur University, Gorakhpur, Uttar Pradesh, India; ^2^Department of Biological Science, The University of Tulsa, Tulsa, OK, United States; ^3^Key Laboratory of Germplasm Enhancement, Physiology and Ecology of Food Crops in Cold Region of Chinese Education Ministry, College of Agriculture, Northeast Agricultural University, Harbin, China; ^4^U.S. Department of Agriculture, Animal and Plant Health Inspection Service, Center for Plant Health Science and Technology, Beltsville, MD, United States; ^5^State Key Laboratory for Biology of Plant Diseases and Insect Pests, Institute of Plant Protection, Chinese Academy of Agricultural Sciences, Beijing, China

**Keywords:** plant virus, epidemiology, detection, interaction, management

Plant viruses have been acknowledged as significant plant diseases since more than a century ago. In recent years, we have gained a greater understanding of the genomes and proteins of plant viruses, as well as how those viruses interact with their hosts. Due to the constant emergence of harmful plant viruses, no reliable or long-term control method is currently available. It is, therefore, necessary to understand plant virus epidemiology to better manage the current viral diseases and develop more effective preventive measures for future epidemics.

In this issue, five research articles were published that focused on a range of topics including evolutionary and management strategies for begomoviruses, metagenomics studies for DNA viruses, host resistance screening, and high-throughput sequencing to reveal new viruses.

Geographical distribution explains the coexistence of the begomovirus population and its satellite molecules, indicating their reliance on accelerating disease severity and spread among economically important crops such as papaya across India (Srivastava et al.). The ongoing evolution of new recombinants and their satellites poses a threat to crop production at an alarming rate, affecting the agroeconomic value and disease control due to the potential for efficient vector transmission, host range extension, and resistance breakdown. However, to gain further knowledge in the field, in-depth investigations into molecular understanding as well as vector-mediated and host-dependent dispersal may help to explain the growing begomovirus illness complex. Uncontrolled evolutionary variety in the DNA genome of a begomovirus, mostly caused by a high frequency of mutational changes, may be the cause of a diverse begomovirus population with a shared host. Additionally, the variable mutation frequency showed that the recombination breakpoints were distributed at random, resulting in a varied distribution of recombinational patterns. Furthermore, this phenomenon makes use of several ways to thwart selection pressure and successfully adapt to surroundings with new hosts. So, the begomovirus and its associated satellites are geographically separated from one another.

RNAi technology should be a quick and efficient method for developing resistance to a number of plant viruses including ChiLCV transcript (Shingote et al.). Furthermore, genome editing using the CRISPR-Cas system has made modifying the host's susceptibility factors possible to establish a resistance to ChiLCV. Through the CRISPR-Cas system, a number of non-essential host susceptibility variables may prove to be more effective targets for building broad-range resistance to plant viruses. Transgenic strategies against ChiLCV appeared to be very promising, but the main barrier to the distribution of transgenic types in India is uncertainty over governmental regulations and popular approval.

The most practical and economical method for preventing any plant viral diseases is host-plant resistance. Although some researchers have previously discovered sources of resistance, the majority of their investigations focused on testing against a single isolate or strain of a plant virus in a specific region. Sayiprathap et al. discovered two pigeon pea genotypes [*Cajanus cajan* (L.) Mill sp.] that are broadly resistant to sterility mosaic disease (SMD). Various findings on the genetics of SMD resistance claim that both susceptibility and resistance are prevalent. According to Sayiprathap et al. reports, a single oligogenic recessive gene controls the resistance to SMD. The focus of cutting-edge research should be on building robust resistance using breeding methods and identifying resistance sources using genomics-assisted breeding.

Lan et al., investigated, eight viruses in the diseased *Paris yunnanensis* plants using high-throughput sequencing (HTS) and reverse transcription polymerase chain reaction (RT-PCR). In their discussion of the viral infections of Paris spp., they emphasize that these viral diseases become a serious problem throughout the development of these medicinal plants in China. Guevara-Rivera et al. reported the second instance of a monopartite begomovirus infecting a non-cultivated plant on the American continent and the first description of a New World (NW) monopartite begomovirus species in Mexico Their findings emphasize the value of metagenomic techniques in comprehending the global virome ecology by highlighting further complexity in the genetic makeup and geographic distribution of monopartite begomoviruses.

The amount of sequence data that is currently available has significantly risen thanks to the rapid development of HTS technology, which has also opened up new possibilities for research into the molecular processes underpinning viral pathogenicity. Controlling plant viral infections requires multidisciplinary epidemiology research.

## Author contributions

All authors listed have made a substantial, direct, and intellectual contribution to the work and approved it for publication.

